# 2-[(*E*)-4-(Dimethyl­amino)­benzyl­idene]indan-1-one

**DOI:** 10.1107/S160053681102664X

**Published:** 2011-07-09

**Authors:** Mohamed Ashraf Ali, Rusli Ismail, Tan Soo Choon, Wan-Sin Loh, Hoong-Kun Fun

**Affiliations:** a Institute for Research in Molecular Medicine, Universiti Sains Malaysia, 11800 USM, Penang, Malaysia; b X-ray Crystallography Unit, School of Physics, Universiti Sains Malaysia, 11800 USM, Penang, Malaysia

## Abstract

In the title compound, C_18_H_17_NO, the dihydro­indene ring system is approximately planar, with a maximum deviation of 0.041 (2) Å. This ring system is almost coplanar with the benzene ring, making a dihedral angle of 5.22 (9)°. In the crystal, inter­molecular C—H⋯O hydrogen bonds link the mol­ecules into chains along the *b* axis.

## Related literature

For the background to dihydro­indene and its derivatives, see: Kohlhagen *et al.* (1998 ▶); Prasad *et al.* (2006 ▶); Tomar *et al.* (2007 ▶); Bhat *et al.* (2005 ▶); Trivedi *et al.* (2007 ▶); Solankee *et al.* (2010 ▶); Liu *et al.* (2003 ▶); Trivedi *et al.* (2008 ▶); Cheng *et al.* (2008 ▶). For a closely related structure, see: Ali *et al.* (2010 ▶).
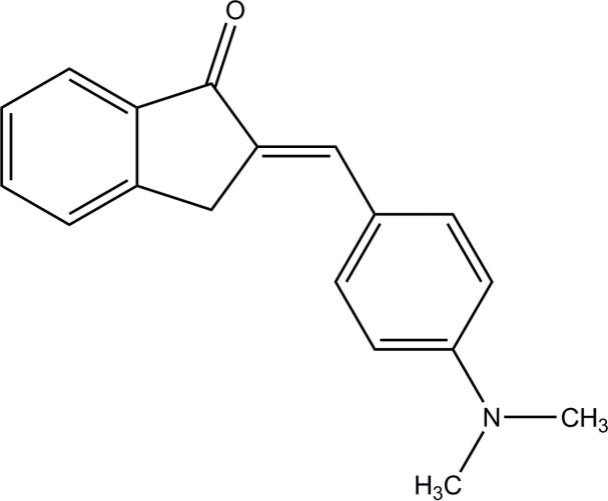


## Experimental

### 

#### Crystal data

C_18_H_17_NO
*M* *_r_* = 263.33
Orthorhombic, 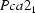

*a* = 30.024 (5) Å
*b* = 5.9898 (9) Å
*c* = 7.6862 (11) Å
*V* = 1382.3 (4) Å^3^
*Z* = 4
Mo *K*α radiation
μ = 0.08 mm^−1^
*T* = 297 K
0.46 × 0.33 × 0.06 mm

#### Data collection

Bruker SMART APEXII DUO CCD area-detector diffractometer
Absorption correction: multi-scan (*SADABS*; Bruker, 2009 ▶) *T*_min_ = 0.965, *T*_max_ = 0.995
8530 measured reflections
2147 independent reflections
1657 reflections with *I* > 2σ(*I*)
*R*_int_ = 0.032

#### Refinement

*R*[*F* ^2^ > 2σ(*F* ^2^)] = 0.037
*wR*(*F* ^2^) = 0.099
*S* = 1.08
2147 reflections
183 parameters
1 restraint
H-atom parameters constrained
Δρ_max_ = 0.12 e Å^−3^
Δρ_min_ = −0.12 e Å^−3^

### 

Data collection: *APEX2* (Bruker, 2009 ▶); cell refinement: *SAINT* (Bruker, 2009 ▶); data reduction: *SAINT*; program(s) used to solve structure: *SHELXTL* (Sheldrick, 2008 ▶); program(s) used to refine structure: *SHELXTL*; molecular graphics: *SHELXTL*; software used to prepare material for publication: *SHELXTL* and *PLATON* (Spek, 2009 ▶).

## Supplementary Material

Crystal structure: contains datablock(s) global, I. DOI: 10.1107/S160053681102664X/wn2441sup1.cif

Structure factors: contains datablock(s) I. DOI: 10.1107/S160053681102664X/wn2441Isup2.hkl

Supplementary material file. DOI: 10.1107/S160053681102664X/wn2441Isup3.cml

Additional supplementary materials:  crystallographic information; 3D view; checkCIF report

## Figures and Tables

**Table 1:** Hydrogen-bond geometry (Å, °)

*D*—H⋯*A*	*D*—H	H⋯*A*	*D*⋯*A*	*D*—H⋯*A*
C9—H9*A*⋯O1^i^	0.97	2.47	3.305 (3)	145

Symmetry code: (i) 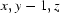.

**Fig. 1.**
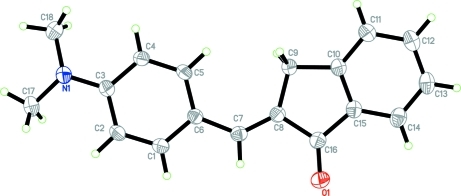
The molecular structure of the title compound, showing 30% probability displacement ellipsoids.

**Fig. 2.**
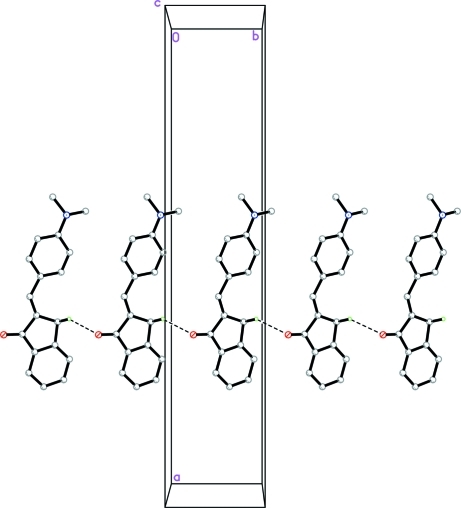
The crystal packing of the title compound, viewed along the c axis. H atoms not involved in the intermolecular interactions (dashed lines) have been omitted for clarity.

## supplementary crystallographic information

### Comment

Novel dihydroindene derivatives are found to be novel Top1 inhibitors with better pharmacokinetic features than camptothecin (CPT). Their moderate biological activity prompted us to investigate their structure activity relationships and a number of the analogs have demonstrated potent cytotoxicity (Kohlhagen *et al.*, 1998). The search for new potent antimicrobial agents with reduced toxicity and lower side effects is a continuous process (Prasad *et al.*, 2006). One of the most frequently encountered groups of organic compounds in medicinal chemistry is dihydroindene its derivatives (Tomar *et al.*, 2007). In addition, dihydroindene derivatives have shown activity against dermatophytes but not against other types of fungi. Dihydroindene derivatives are readily synthesized by the base-catalysed Claisen-Schmidt condensation of an aldehyde and an appropriate ketone in a polar solvent such as ethanol and yields may be variable, ranging from 5% to 80% (Tomar *et al.*, 2007). The dihydroindene derivatives have a diverse range of biological activities, among which antimalarial, antitubercular, anti-inflammatory, cytotoxic, antioxidant, analgesic, antiviral and antimicrobial properties have been widely cited (Tomar *et al.*, 2007; Bhat *et al.*, 2005; Trivedi *et al.*, 2007; Solankee *et al.*, 2010; Liu *et al.*, 2003; Trivedi *et al.*, 2008; Cheng *et al.*, 2008).

In the title compound (Fig. 1), the dihydroindene ring system (C8–C16) is approximately planar, with a maximum deviation of 0.041 (2) Å at atom C15. This ring system is almost coplanar with the benzene ring (C1–C6), with a dihedral angle of 5.22 (9)°. Bond lengths and angles are within the normal ranges and are comparable to those in the related crystal structure (Ali *et al.*, 2010).

In the crystal packing (Fig. 2), intermolecular C9—H9A···O1 hydrogen bonds (Table 1) link the molecules into chains along the *b* axis.

### Experimental

A mixture of 2,3-dihydro-1*H*-indene-1-one (0.001 mmol) and 4-nitrobenzaldehyde (0.001 mmol) was dissolved in methanol (10 ml) and to this mixture was added 30% sodium hydroxide solution (5 ml). The mixture was stirred for 5 h. After the completion of the reaction, as evident from TLC, the mixture was poured on to crushed ice, then neutralized with concentrated HCl. The precipitated solid was filtered, washed with water and recrystallized from ethanol to yield the title compound as light yellow crystals.

### Refinement

All H atoms were positioned geometrically and refined using a riding model with *U*_iso_(H) = 1.2 or 1.5 *U*_eq_(C) [C–H = 0.93–0.97 Å]. A rotating group model was applied to the methyl groups. In the absence of significant anomalous scattering effects, 1725 Friedel pairs were merged for the final refinement.

### Crystal data

**Table:**

C_18_H_17_NO	*F*(000) = 560
*M**_r_* = 263.33	*D*_x_ = 1.265 Mg m^−^^3^
Orthorhombic, *P**c**a*2_1_	Mo *K*α radiation, λ = 0.71073 Å
Hall symbol: P 2c -2ac	Cell parameters from 2132 reflections
*a* = 30.024 (5) Å	θ = 2.7–23.6°
*b* = 5.9898 (9) Å	µ = 0.08 mm^−^^1^
*c* = 7.6862 (11) Å	*T* = 297 K
*V* = 1382.3 (4) Å^3^	Plate, yellow
*Z* = 4	0.46 × 0.33 × 0.06 mm

### Data collection

**Table:**

Bruker SMART APEXII DUO CCD area-detector diffractometer	2147 independent reflections
Radiation source: fine-focus sealed tube	1657 reflections with *I* > 2σ(*I*)
graphite	*R*_int_ = 0.032
φ and ω scans	θ_max_ = 30.0°, θ_min_ = 2.7°
Absorption correction: multi-scan (*SADABS*; Bruker, 2009)	*h* = −42→42
*T*_min_ = 0.965, *T*_max_ = 0.995	*k* = −7→8
8530 measured reflections	*l* = −10→10

### Refinement

**Table:**

Refinement on *F*^2^	Primary atom site location: structure-invariant direct methods
Least-squares matrix: full	Secondary atom site location: difference Fourier map
*R*[*F*^2^ > 2σ(*F*^2^)] = 0.037	Hydrogen site location: inferred from neighbouring sites
*wR*(*F*^2^) = 0.099	H-atom parameters constrained
*S* = 1.08	*w* = 1/[σ^2^(*F*_o_^2^) + (0.0455*P*)^2^ + 0.062*P*] where *P* = (*F*_o_^2^ + 2*F*_c_^2^)/3
2147 reflections	(Δ/σ)_max_ = 0.001
183 parameters	Δρ_max_ = 0.12 e Å^−^^3^
1 restraint	Δρ_min_ = −0.12 e Å^−^^3^

### Special details

**Table:**

Geometry. All e.s.d.'s (except the e.s.d. in the dihedral angle between two l.s. planes) are estimated using the full covariance matrix. The cell e.s.d.'s are taken into account individually in the estimation of e.s.d.'s in distances, angles and torsion angles; correlations between e.s.d.'s in cell parameters are only used when they are defined by crystal symmetry. An approximate (isotropic) treatment of cell e.s.d.'s is used for estimating e.s.d.'s involving l.s. planes.
Refinement. Refinement of *F*^2^ against ALL reflections. The weighted *R*-factor *wR* and goodness of fit *S* are based on *F*^2^, conventional *R*-factors *R* are based on *F*, with *F* set to zero for negative *F*^2^. The threshold expression of *F*^2^ > σ(*F*^2^) is used only for calculating *R*-factors(gt) *etc*. and is not relevant to the choice of reflections for refinement. *R*-factors based on *F*^2^ are statistically about twice as large as those based on *F*, and *R*- factors based on ALL data will be even larger.

### Fractional atomic coordinates and isotropic or equivalent isotropic displacement parameters (Å^2^)

**Table:**

	*x*	*y*	*z*	*U*_iso_*/*U*_eq_	
O1	0.33515 (5)	0.7381 (2)	0.9621 (3)	0.0591 (4)	
N1	0.58774 (5)	0.0911 (3)	0.8681 (3)	0.0523 (4)	
C1	0.49445 (7)	0.4669 (3)	0.9738 (3)	0.0466 (5)	
H1A	0.4900	0.6005	1.0329	0.056*	
C2	0.53678 (7)	0.3789 (3)	0.9663 (3)	0.0474 (5)	
H2A	0.5601	0.4536	1.0207	0.057*	
C3	0.54532 (6)	0.1784 (3)	0.8779 (3)	0.0409 (4)	
C4	0.50852 (6)	0.0695 (3)	0.8021 (3)	0.0428 (4)	
H4A	0.5127	−0.0657	0.7449	0.051*	
C5	0.46634 (6)	0.1601 (3)	0.8111 (3)	0.0418 (4)	
H5A	0.4428	0.0843	0.7595	0.050*	
C6	0.45795 (6)	0.3632 (3)	0.8959 (2)	0.0391 (4)	
C7	0.41495 (6)	0.4734 (3)	0.9089 (3)	0.0414 (4)	
H7A	0.4157	0.6095	0.9671	0.050*	
C8	0.37449 (6)	0.4142 (3)	0.8522 (3)	0.0405 (4)	
C9	0.35932 (6)	0.2075 (3)	0.7567 (3)	0.0443 (4)	
H9A	0.3664	0.0736	0.8221	0.053*	
H9B	0.3731	0.1979	0.6427	0.053*	
C10	0.30926 (6)	0.2391 (3)	0.7417 (3)	0.0426 (4)	
C11	0.27778 (6)	0.0936 (4)	0.6759 (3)	0.0502 (5)	
H11A	0.2863	−0.0435	0.6298	0.060*	
C12	0.23334 (7)	0.1555 (4)	0.6796 (3)	0.0556 (5)	
H12A	0.2119	0.0594	0.6343	0.067*	
C13	0.22019 (7)	0.3589 (4)	0.7499 (3)	0.0572 (5)	
H13A	0.1902	0.3971	0.7518	0.069*	
C14	0.25135 (7)	0.5039 (3)	0.8169 (3)	0.0536 (5)	
H14A	0.2427	0.6401	0.8643	0.064*	
C15	0.29614 (6)	0.4420 (3)	0.8119 (3)	0.0431 (4)	
C16	0.33507 (6)	0.5593 (3)	0.8853 (3)	0.0433 (4)	
C17	0.62544 (7)	0.2311 (4)	0.9114 (4)	0.0648 (7)	
H17A	0.6243	0.2695	1.0326	0.097*	
H17B	0.6245	0.3649	0.8426	0.097*	
H17C	0.6526	0.1519	0.8876	0.097*	
C18	0.59698 (7)	−0.0926 (4)	0.7520 (4)	0.0626 (6)	
H18A	0.5777	−0.2155	0.7792	0.094*	
H18B	0.6274	−0.1382	0.7654	0.094*	
H18C	0.5920	−0.0462	0.6340	0.094*	

### Atomic displacement parameters (Å^2^)

**Table:**

	*U*^11^	*U*^22^	*U*^33^	*U*^12^	*U*^13^	*U*^23^
O1	0.0587 (9)	0.0423 (8)	0.0762 (11)	0.0033 (6)	0.0014 (8)	−0.0079 (8)
N1	0.0402 (8)	0.0582 (10)	0.0585 (11)	0.0032 (7)	−0.0044 (8)	−0.0105 (9)
C1	0.0468 (11)	0.0445 (10)	0.0485 (11)	−0.0034 (8)	−0.0011 (9)	−0.0097 (9)
C2	0.0422 (10)	0.0517 (11)	0.0484 (11)	−0.0068 (8)	−0.0055 (9)	−0.0099 (10)
C3	0.0397 (9)	0.0456 (9)	0.0375 (9)	−0.0028 (7)	−0.0002 (8)	0.0006 (8)
C4	0.0441 (10)	0.0387 (9)	0.0457 (11)	−0.0029 (7)	−0.0001 (8)	−0.0041 (8)
C5	0.0394 (9)	0.0399 (9)	0.0462 (10)	−0.0074 (7)	−0.0027 (8)	−0.0035 (8)
C6	0.0391 (9)	0.0397 (9)	0.0384 (10)	−0.0046 (7)	0.0014 (8)	0.0010 (8)
C7	0.0459 (10)	0.0365 (9)	0.0419 (11)	−0.0027 (7)	0.0037 (8)	0.0009 (8)
C8	0.0404 (9)	0.0376 (9)	0.0437 (10)	−0.0016 (7)	0.0037 (8)	0.0023 (8)
C9	0.0388 (9)	0.0434 (9)	0.0507 (11)	−0.0009 (7)	0.0016 (8)	−0.0020 (9)
C10	0.0400 (9)	0.0463 (10)	0.0415 (10)	−0.0026 (7)	−0.0001 (8)	0.0061 (9)
C11	0.0499 (11)	0.0533 (11)	0.0474 (11)	−0.0051 (9)	−0.0020 (9)	0.0017 (10)
C12	0.0455 (11)	0.0691 (14)	0.0523 (12)	−0.0102 (10)	−0.0053 (10)	0.0080 (12)
C13	0.0401 (10)	0.0709 (14)	0.0606 (13)	0.0029 (9)	−0.0030 (10)	0.0141 (12)
C14	0.0477 (10)	0.0536 (10)	0.0597 (13)	0.0070 (9)	0.0015 (10)	0.0096 (11)
C15	0.0414 (9)	0.0437 (9)	0.0442 (10)	−0.0005 (7)	0.0016 (8)	0.0078 (9)
C16	0.0451 (10)	0.0373 (9)	0.0476 (11)	0.0003 (7)	0.0037 (9)	0.0072 (9)
C17	0.0396 (10)	0.0734 (15)	0.0815 (18)	−0.0012 (10)	−0.0097 (11)	−0.0083 (14)
C18	0.0513 (12)	0.0626 (13)	0.0738 (16)	0.0101 (10)	0.0024 (12)	−0.0112 (13)

### Geometric parameters (Å, °)

**Table:**

O1—C16	1.223 (2)	C9—H9A	0.9700
N1—C3	1.379 (2)	C9—H9B	0.9700
N1—C18	1.444 (3)	C10—C11	1.382 (3)
N1—C17	1.448 (3)	C10—C15	1.387 (3)
C1—C2	1.377 (3)	C11—C12	1.385 (3)
C1—C6	1.394 (3)	C11—H11A	0.9300
C1—H1A	0.9300	C12—C13	1.390 (3)
C2—C3	1.403 (3)	C12—H12A	0.9300
C2—H2A	0.9300	C13—C14	1.377 (3)
C3—C4	1.409 (3)	C13—H13A	0.9300
C4—C5	1.380 (2)	C14—C15	1.396 (3)
C4—H4A	0.9300	C14—H14A	0.9300
C5—C6	1.403 (3)	C15—C16	1.476 (3)
C5—H5A	0.9300	C17—H17A	0.9600
C6—C7	1.454 (2)	C17—H17B	0.9600
C7—C8	1.339 (2)	C17—H17C	0.9600
C7—H7A	0.9300	C18—H18A	0.9600
C8—C16	1.490 (2)	C18—H18B	0.9600
C8—C9	1.510 (3)	C18—H18C	0.9600
C9—C10	1.519 (3)		
			
C3—N1—C18	120.01 (17)	C11—C10—C15	120.07 (18)
C3—N1—C17	119.35 (17)	C11—C10—C9	128.74 (18)
C18—N1—C17	115.67 (18)	C15—C10—C9	111.15 (16)
C2—C1—C6	122.48 (19)	C10—C11—C12	118.9 (2)
C2—C1—H1A	118.8	C10—C11—H11A	120.6
C6—C1—H1A	118.8	C12—C11—H11A	120.6
C1—C2—C3	121.07 (17)	C11—C12—C13	121.1 (2)
C1—C2—H2A	119.5	C11—C12—H12A	119.5
C3—C2—H2A	119.5	C13—C12—H12A	119.5
N1—C3—C2	121.30 (16)	C14—C13—C12	120.4 (2)
N1—C3—C4	121.75 (17)	C14—C13—H13A	119.8
C2—C3—C4	116.95 (17)	C12—C13—H13A	119.8
C5—C4—C3	121.12 (18)	C13—C14—C15	118.5 (2)
C5—C4—H4A	119.4	C13—C14—H14A	120.8
C3—C4—H4A	119.4	C15—C14—H14A	120.8
C4—C5—C6	121.96 (16)	C10—C15—C14	121.14 (18)
C4—C5—H5A	119.0	C10—C15—C16	109.94 (16)
C6—C5—H5A	119.0	C14—C15—C16	128.78 (19)
C1—C6—C5	116.40 (16)	O1—C16—C15	127.09 (17)
C1—C6—C7	117.79 (17)	O1—C16—C8	126.27 (17)
C5—C6—C7	125.81 (16)	C15—C16—C8	106.62 (16)
C8—C7—C6	131.55 (17)	N1—C17—H17A	109.5
C8—C7—H7A	114.2	N1—C17—H17B	109.5
C6—C7—H7A	114.2	H17A—C17—H17B	109.5
C7—C8—C16	120.69 (17)	N1—C17—H17C	109.5
C7—C8—C9	130.51 (16)	H17A—C17—H17C	109.5
C16—C8—C9	108.78 (15)	H17B—C17—H17C	109.5
C8—C9—C10	103.47 (15)	N1—C18—H18A	109.5
C8—C9—H9A	111.1	N1—C18—H18B	109.5
C10—C9—H9A	111.1	H18A—C18—H18B	109.5
C8—C9—H9B	111.1	N1—C18—H18C	109.5
C10—C9—H9B	111.1	H18A—C18—H18C	109.5
H9A—C9—H9B	109.0	H18B—C18—H18C	109.5
			
C6—C1—C2—C3	0.3 (3)	C8—C9—C10—C15	2.3 (2)
C18—N1—C3—C2	−169.1 (2)	C15—C10—C11—C12	0.7 (3)
C17—N1—C3—C2	−15.2 (3)	C9—C10—C11—C12	178.2 (2)
C18—N1—C3—C4	11.7 (3)	C10—C11—C12—C13	−0.7 (3)
C17—N1—C3—C4	165.7 (2)	C11—C12—C13—C14	0.3 (4)
C1—C2—C3—N1	179.1 (2)	C12—C13—C14—C15	0.1 (3)
C1—C2—C3—C4	−1.7 (3)	C11—C10—C15—C14	−0.3 (3)
N1—C3—C4—C5	−179.24 (19)	C9—C10—C15—C14	−178.2 (2)
C2—C3—C4—C5	1.6 (3)	C11—C10—C15—C16	175.67 (19)
C3—C4—C5—C6	−0.2 (3)	C9—C10—C15—C16	−2.2 (2)
C2—C1—C6—C5	1.1 (3)	C13—C14—C15—C10	−0.1 (3)
C2—C1—C6—C7	−178.73 (19)	C13—C14—C15—C16	−175.3 (2)
C4—C5—C6—C1	−1.2 (3)	C10—C15—C16—O1	−177.3 (2)
C4—C5—C6—C7	178.63 (19)	C14—C15—C16—O1	−1.7 (4)
C1—C6—C7—C8	−178.3 (2)	C10—C15—C16—C8	1.1 (2)
C5—C6—C7—C8	1.8 (3)	C14—C15—C16—C8	176.7 (2)
C6—C7—C8—C16	179.24 (19)	C7—C8—C16—O1	0.4 (3)
C6—C7—C8—C9	1.3 (4)	C9—C8—C16—O1	178.8 (2)
C7—C8—C9—C10	176.6 (2)	C7—C8—C16—C15	−178.01 (18)
C16—C8—C9—C10	−1.5 (2)	C9—C8—C16—C15	0.4 (2)
C8—C9—C10—C11	−175.3 (2)		

### Hydrogen-bond geometry (Å, °)

**Table:**

*D*—H···*A*	*D*—H	H···*A*	*D*···*A*	*D*—H···*A*
C9—H9A···O1^i^	0.97	2.47	3.305 (3)	145

Symmetry codes: (i) *x*, *y*−1, *z*.
